# Supplementary material for: Individual Work Attitudes and Work Ability
Source: Eur J Investig Health Psychol Educ. 2025 Apr 3;15(4):53. doi: 10.3390/ejihpe15040053 (PMC12025400; doi:10.3390/ejihpe15040053)
Supplement: Supplementary file 1 [file ejihpe-15-00053-s001.zip › ejihpe-3504056-supplementary.pdf]

SUPPLEMENTARY TABLE S1

Table S1. Comparison of standardized estimates obtained with the Ordinary Least Squares (OLS) and robust methods for hierarchical regression models

Model	Predictor	OLS	Robust	OLS VIF
Model I	Sex (Female)	0.013 [–0.041; 0.067]	0.009 [–0.037; 0.055]	1.021
	Age	–0.078 [–0.133; –0.023]	–0.063 [–0.109; –0.016]	1.043
	Work Annoyance	–0.187 [–0.243; –0.131]	–0.201 [–0.248; –0.154]	1.064
Model II	Sex (Female)	0.019 [–0.035; 0.073]	0.017 [–0.029; 0.064]	1.023
	Age	–0.052 [–0.108; 0.003]	–0.041 [–0.089; 0.007]	1.075
	Work Annoyance	–0.160 [–0.216; –0.103]	–0.179 [–0.227; –0.130]	1.102
	Social Capital	0.149 [0.093; 0.205]	0.133 [0.085; 0.182]	1.089
Model III	Sex (Female)	0.020 [–0.033; 0.074]	0.021 [–0.025; 0.067]	1.023
	Age	–0.043 [–0.098; 0.012]	–0.037 [–0.085; 0.011]	1.081
	Work Annoyance	–0.143 [–0.200; –0.087]	–0.169 [–0.218; –0.120]	1.119
	Social Capital	0.116 [0.058; 0.173]	0.107 [0.057; 0.156]	1.159
	Overcommitment	–0.132 [–0.188; –0.075]	–0.103 [–0.152; –0.054]	1.123
Model IV[§]	Sex (Female)	–0.005 [–0.056; 0.045]	–0.004 [–0.050; 0.042]	1.031
	Age	–0.053 [–0.105; –0.001]	–0.049 [–0.096; –0.002]	1.091
	Work Annoyance	–0.010 [–0.067; 0.048]	–0.017 [–0.067; 0.033]	1.313
	Social Capital	–0.003 [–0.059; 0.054]	0.007 [–0.043; 0.057]	1.290
	Overcommitment	–0.090 [–0.146; –0.035]	–0.088 [–0.136; –0.039]	1.211
	Vigor	0.234 [0.151; 0.317]	0.207 [0.145; 0.269]	2.723
	Dedication	0.166 [0.085; 0.246]	0.157 [0.096; 0.218]	2.599
	Absorption	0.020 [–0.050; 0.089]	0.034 [–0.022; 0.090]	1.964

(continues)

Model	Predictor	OLS	Robust	OLS VIF
Model V	Sex (Female)	0.015 [–0.039; 0.070]	0.014 [–0.032; 0.061]	1.026
	Age	–0.055 [–0.111; 0.001]	–0.043 [–0.091; 0.005]	1.076
	Work Annoyance	–0.156 [–0.213; –0.100]	–0.178 [–0.226; –0.129]	1.105
	Social Capital	0.155 [0.099; 0.211]	0.143 [0.095; 0.191]	1.094
	Work Annoyance × Social Capital	0.071 [0.019; 0.122]	0.071 [0.027; 0.115]	1.011
Model VI§	Sex (Female)	–0.009 [–0.060; 0.042]	–0.006 [–0.052; 0.039]	1.033
	Age	–0.055 [–0.108; –0.003]	–0.051 [–0.097; –0.004]	1.092
	Work Annoyance	–0.007 [–0.064; 0.051]	–0.015 [–0.065; 0.034]	1.316
	Social Capital	0.003 [–0.054; 0.060]	0.013 [–0.037; 0.062]	1.299
	Overcommitment	–0.092 [–0.147; –0.036]	–0.088 [–0.136; –0.040]	1.211
	Vigor	0.229 [0.146; 0.312]	0.201 [0.140; 0.261]	2.732
	Dedication	0.165 [0.084; 0.246]	0.156 [0.097; 0.215]	2.600
	Absorption	0.026 [–0.045; 0.096]	0.040 [–0.015; 0.095]	1.973
	Work Annoyance × Social Capital	0.059 [0.011; 0.107]	0.055 [0.011; 0.098]	1.018

Note: §: the robust estimates have been obtained with ridge regression

SUPPLEMENTARY TABLE S2

Table S2. Comparison of standardized estimates obtained with the Ordinary Least Squares (OLS) and robust methods for hierarchical regression models including night work as additional predictor

**Model**	**Predictor**	**OLS**	**Robust**	**OLS VIF**
Model IV§	Sex (Female)	–0.009 [–0.060; 0.042]	–0.007 [–0.053; 0.039]	1.034
	Age	–0.055 [–0.107; –0.002]	–0.051 [–0.097; –0.004]	1.091
	Work Annoyance	–0.013 [–0.071; 0.045]	–0.020 [–0.070; 0.030]	1.316
	Social Capital	–0.006 [–0.063; 0.051]	0.005 [–0.045; 0.055]	1.293
	Overcommitment	–0.086 [–0.142; –0.031]	–0.084 [–0.132; –0.036]	1.217
	Vigor	0.226 [0.143; 0.309]	0.199 [0.139; 0.260]	2.743
	Dedication	0.173 [0.092; 0.254]	0.161 [0.101; 0.221]	2.608
	Absorption	0.022 [–0.049; 0.092]	0.037 [–0.019; 0.093]	1.965
	Night work	–0.060 [–0.111; –0.009]	–0.055 [–0.101; –0.010]	1.024
Model VI§	Sex (Female)	–0.012 [–0.063; 0.039]	–0.009 [–0.054; 0.036]	1.037
	Age	–0.057 [–0.109; –0.004]	–0.052 [–0.098; –0.006]	1.092
	Work Annoyance	–0.010 [–0.067; 0.048]	–0.018 [–0.067; 0.030]	1.319
	Social Capital	0.000 [–0.057; 0.057]	0.011 [–0.038; 0.059]	1.302
	Overcommitment	–0.087 [–0.143; –0.032]	–0.084 [–0.131; –0.037]	1.217
	Vigor	0.221 [0.138; 0.304]	0.193 [0.135; 0.251]	2.750
	Dedication	0.171 [0.090; 0.252]	0.158 [0.101; 0.215]	2.609
	Absorption	0.027 [–0.043; 0.098]	0.042 [–0.012; 0.096]	1.974
	Night work	–0.057 [–0.108; –0.007]	–0.053 [–0.098; –0.008]	1.026
	Work Annoyance × Social Capital	0.057 [0.009; 0.105]	0.052 [0.009; 0.095]	1.019

Note: §: the robust estimates have been obtained with ridge regression
